# Enhancement of dispersion stability of inorganic additives via poly(sodium-4-styrenesulfonate) treatment geared to hydrogel applications

**DOI:** 10.3906/kim-2105-62

**Published:** 2021-10-19

**Authors:** Filiz BORAN, Merve OKUTAN

**Affiliations:** 1 Department of Chemical Engineering, Faculty of Engineering, Hitit University, Çorum Turkey

**Keywords:** Bentonite, clinoptilolite, poly(sodium 4-styrenesulfonate), polyvinylalcohol, polyvinylpyrrolidone, rhodamine B

## Abstract

This study reports the preparation of poly(sodium-4-styrene sulfonate) (PSS) treated bentonite and clinoptilolite to prevent the agglomeration and sedimentation of these inorganic fillers during the preparation of hydrogel. For this purpose PSS treated fillers were prepared by using various techniques (dip and dry, hydrothermal, one-step ball milling and ultrasonication methods). The most suitable technique for preparing these PSS treated inorganic fillers (abbreviated as BP-dip and CP-dip) was the dip and dry method. BP-dip and CP-dip based polyvinyl alcohol/polyvinylpyrrolidone (PVA/PVP) composite hydrogels were prepared using the freeze/thawing method after the addition of one of BP-dip and CP-dip inorganic fillers in various amounts. The swelling properties, stability behaviors and Rhodamine B (RhB) adsorption of the composite hydrogels were studied. It was found that the swelling degrees of CP-dip and BP-dip based composite hydrogels with 25 mg of filler were higher than that of all other samples. The kinetic mechanism of RhB adsorption process and the related characteristic kinetic parameters were investigated by Pseudo kinetic models. The adsorption kinetics results for RhB adsorption were found best fitted with pseudo-second-order kinetics model. The maximum RhB adsorption capacity was determined to be for PVA/PVP-CP-dip25, which was 3.3 times higher than that of the unfilled PVA/PVP hydrogel.

## 1. Introduction

Hydrogels are crosslinked, three-dimensional polymers which have high swelling capacity, softness, hydrophilic and nonsoluble characteristics [1–4]. They stand out as effective materials in the treatment of various aqueous pollutants [2]. Polyvinyl alcohol (PVA) and polyvinylpyrrolidone (PVP) are the most widely used polymers to prepare hydrogels due to their physical and chemical properties, such as low toxicity, biocompatibility and biodegradability, high water solubility, thermostability, chemical resistivity and gel-forming ability [5–10]. By preparing PVA/PVP blend hydrogels, the properties of the two polymer components can be advantageously combined [6, 10]. For example, the preparation of PVA-based hydrogels with added PVP polymer improves the mechanical properties of the resulting hydrogel and decreases the friction coefficient [6].

One of the most important problems of the present century is water pollution and a decrease of clean water resources. Water pollution mainly originates from contamination by several chemicals, including toxic metal ions and synthetic organic chemicals, like dyes [11]. To remove the pollutants studies on the development of adsorbents with polymeric matrices to provide economical and efficient treatment have increased in recent years. One of the most common forms of these polymeric materials is hydrogels. Hydrogels are hydrophilic materials that have significant swelling and water retention properties. But, they should not dissolve in water and need to have good hydrolytic degradation properties. To use polymeric hydrogels effectively in water treatment, their stability in water must be high. Hydrogel stability can be improved by the use of various inorganic additives, such as montmorillonite, halloysite, mica, etc. These additives can enhance the mechanical and chemical stabilities of the hydrogels in addition to their adsorption capabilities [12–14]. Hydrogels containing a large number of various filler materials, such as ZnO-TiO2 [15], halloysite [13], montmorillonite [16], graphene oxide [17] composites have been described in the literature for improving their performances and properties on oil-absorbent, a piezoresistive pressure sensor, mechanical strength, water stability, swelling properties, the adsorption of ionic molecules and the removal of organic dyes, such as methylene blue and congo red [2, 18]. The filler materials are required to have good dispersibility in the polymer matrix for these applications. For this purpose the fillers, for instance halloysite, montmorillonite, ZnO-TiO2 and graphene oxide, need to be uniformly distributed in an aqueous medium or in a polymeric hydrogel with new and cheaper surface modification methods. Several methods have been reported for the modification of clays, including cold plasma techniques, hydrothermal, surfactant modification and physical impregnation techniques and activation by sodium ions, calcium ions, heating and acids for enhancing their adsorption and catalytic properties [18, 19–22]. In addition, a wide variety of filler modification methods, such as thermomechanical treatment technique, one-step in situ ball milling, hydrothermal autoclaving and dip and dry method, etc. have been applied to various other fillers, such as carbon black, hydroxyapatite and various other inorganic material as described in the literature [23–26]. However, as far as we know, studies applying these latter methods to clays have not been reported yet. Therefore, various simpler, cheaper and environmentally friendly methods need to be studied for surface modification of clays.

In this study clinoptilolite and bentonite were used as hydrogel additives. To prepare homogeneous hydrogels both fillers, which tend to precipitate in aqueous media, needed to be uniformly distributed in the polymer’s matrix. For this purpose the clinoptilolite and bentonite were surface treated with PSS to increase their hydrophilicity and provide the needed good dispersibility in water and/or hydrogels. PSS is a simple polyelectrolyte used in the synthesis and modification of various inorganic materials to help control material size and morphology [27–29]. Four different techniques, a dip and dry method, ball milling, ultrasound and hydrothermal, were used for the treatment of the fillers with PSS and the resultant materials were compared for use as hydrogel fillings. In addition, the PSS treated filler based PVA/PVP composite hydrogels were prepared by a freeze/thawing method. Structural properties, morphological properties and adsorption behavior of RhB of these composite hydrogels were investigated relative to bare hydrogels.

## 2. Materials and methods

### 2.1. Materials

Bentonite, PVA (Mw: 145,000), N,N-dimethylformamide (DMF) and RhB were supplied by Merck (Turkey). Clinoptilolite was obtained from Gördes region (Manisa, Turkey). PSS was provided by Aldrich (Turkey) (Mw: 70,000). PVP was purchased from Fluka (Turkey) (Mw: 40,000). The clinoptilolite was separated according to grain size by sieving, washed with distilled water until completely clear and finally dried at 105 °C. The other chemicals were used without further purification.

### 2.2. PSS treatment of bentonite and clinoptilolite

Four different methods were used for treatment of the bentonite and clinoptilolite with PSS. To prepare PSS coated bentonite and clinoptilolite by the dip and dry method, 0.2 g of the inorganic filler was dispersed in 30 mL of DMF. Then this solution was added to 10 mL of PSS (2.1 g) solution and stirred at 0 °C for 2 h. The precipitate was filtered and dried at 110 °C for 12 h and the drying procedure was repeated five times as a cooling-heating cycle to completely remove the DMF [26]. For use in the other three treatment processes 2.5 g of bentonite (or clinoptilolite) was dispersed in 100 mL of PSS (2.5 g) aqueous solution in different flasks. For the ultrasound, ball-milling [25] and hydrothermal processes [24], the surface treatment was performed with an ultrasonic bath at 80 °C, with planetary ball milling at a certain frequency (30 Hz-speed) and with an autoclave at 150 °C for 2 h, in the PSS solution mentioned above respectively. Then each product was filtered and dried at 110 °C for 12 h. The samples were labeled as in Table 1.

**Table 1:** Abbreviations for untreated and PSS treated inorganic fillers according to their preparation technique.

Bentonite		Clinoptilolite	
Preparation technique	Abb.	Preparation technique	Abb.
Untreated bentonite	B	Untreated clinoptilolite	C
Ball milling (untreated bentonite)	B-ball	Ball milling (untreated clinoptilolite)	C-ball
Ball milling (PSS treated bentonite)	BP-ball	Ball milling (PSS treated clinoptilolite)	CP-ball
Dip and dry method (PSS treated bentonite)	BP-dip	Dip and dry method (PSS treated clinoptilolite)	CP-dip
Ultrasound (PSS treated bentonite)	BP-ult	Ultrasound (PSS treated clinoptilolite)	CP-ult
Hydrothermal (PSS treated bentonite)	BP-hyd	Hydrothermal (PSS treated clinoptilolite)	CP-hyd

### 2.3. Synthesis of bentonite and clinoptilolite based PVA/PVP hydrogels

**Figure 1:**
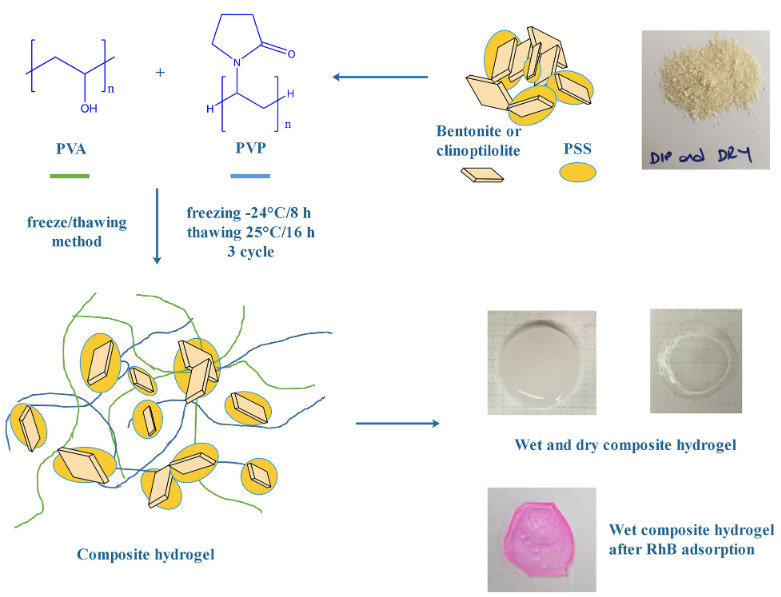
Schematic representation of composite hydrogel preparation steps and synthesized hydrogels photos (wet, dry and dye adsorbed).

The bentonite and clinoptilolite based PVA/PVP hydrogels were synthesized using the freeze/thawing method (Figure 1). First of all, a PVA aqueous solution (5 wt %) was prepared at 90 ºC for 2 h by stirring and then cooled down to room temperature. Then it was mixed with a PVP aqueous solution (5 wt %) at room temperature with stirring overnight. This polymers mixture was poured into a well plate and a freezing/thawing cycle was performed to obtain the hydrogel. The freezing and thawing temperatures/times were chosen as –24 °C/8 h and 25 °С/16 h, respectively. This process was repeated three times. The hydrogels were then washed four times by soaking in distilled water for two days to remove unreacted polymers by changing the washing water. Finally, they were dried at room temperature. The same steps were repeated after adding 25, 50 and 100 mg of filler to the initial polymer mixture to make the bentonite and clinoptilolite based hydrogels. The samples were labeled as in Table 2.

**Table 2:** Abbreviations for composite hydrogels according to filler type and amount.

Bentonite based hydrogels		Clinoptilolite based hydrogels	
Sample name	Abb.	Sample name	Abb.
Bare PVA/PVP hydrogel	PVA/PVP	Bare PVA/PVP hydrogel	PVA/PVP
Bentonite (25 mga)based PVA/PVP hydrogel	PVA/PVP-B25	Clinoptilolite (25 mg)based PVA/PVP hydrogel	PVA/PVP-C25
PSS treated bentonite (25 mg)based PVA/PVP hydrogel	PVA/PVP-BP-dip25	PSS treated clinoptilolite (25 mg)based PVA/PVP hydrogel	PVA/PVP-CPdip25
Bentonite (50 mg)based PVA/PVP hydrogel	PVA/PVP-B50	Clinoptilolite (50 mg)based PVA/PVP hydrogel	PVA/PVP-C50
PSS treated bentonite (50 mg)based PVA/PVP hydrogel	PVA/PVP-BP-dip50	PSS treated clinoptilolite (50 mg)based PVA/PVP hydrogel	PVA/PVP-CP-dip50
Bentonite (100 mg)based PVA/PVP hydrogel	PVA/PVP-B100	Clinoptilolite (100 mg)based PVA/PVP hydrogel	PVA/PVP-C100
PSS treated bentonite (100 mg)based PVA/PVP hydrogel	PVA/PVP-BP-dip100	PSS treated clinoptilolite (100 mg)based PVA/PVP hydrogel	PVA/PVP-CP-dip100

a weights of filler are per 5 g of PVA/PVP.

### 2.4. Characterization

The Fourier transform infrared spectra (FTIR) of the PSS treated bentonite and clinoptilolite were obtained by a Thermo Scientific Nicolet 6700 FT-IR spectrometer equipped with an ATR (attenuated total reflectance) attachment in the wave number range from 600 to 4000 cm^–^1. The zeta potentials of the untreated and PSS treated bentonite and clinoptilolite were determined by a ZetaSizer Nano ZSP laser particle analyzer (Malvern). Elemental analysis of PSS and the BP-dip and CP-dip particles was performed with an energy dispersive X-ray (EDX) device integrated into an EVO 40 Model scanning electron microscope (SEM, ZEISS) device in Ankara University Institute of Nuclear Sciences. The surface morphology of the PSS treated inorganic additives and the composite hydrogels were characterized by field emission scanning electron microscope with FEG electron-induced (FESEM, Quanta 450 FEG model, 20-30 kV, FEI). The samples, dried at room temperature, were covered with a thin layer of gold before being analyzed with the FESEM. UV-Vis adsorption experiments were carried out using a Genesys 10S UV-Visible (Thermo Scientific, USA) spectrophotometer. The FESEM, FTIR and zeta potential analyses were conducted at Hitit University Scientific Technical Application and Research Center (HÜBTUAM), Çorum, Turkey.

### 2.5. Swelling studies

The swelling studies were performed at room temperature using the dried samples which were then soaked in distilled water for 24 h. The swollen hydrogels were taken out, wiped and weighed. The swelling degree of the hydrogels (SD%) was determined based on Eq. 1:

(1)SD(%)=Ws-WidWidx100

where w_id_ and w_s_ are the masses of initial dry hydrogel and swollen hydrogel, respectively.

The solution stabilities of all of the hydrogels were appraised by polymer mass loss in buffer solution at pH 6.5 for 15 days. The masses of each hydrogel were weighed after 1 day, 2 days, 4 days and 15 days of immersion. The immersed hydrogels were wiped with a filter paper to remove the excess water on the surface before weighing. At the end of immersion for 15 days, the immersed hydrogels were dried to a constant weight at 50 °C (w_fd_) [6,30]. The percentage of hydrogel mass losses was calculated with the Eq. 2:

(2)Mass loss (%) =Wid-WfdWidx100

where w_id_ and w_fd_ are the masses of initial dry hydrogel and final dry hydrogels, respectively.

### 2.6. Adsorption studies

For the adsorption properties of RhB, 50 mg of dry hydrogels were immersed in 50 mL of 5 mg/L RhB solution and agitated on a mechanical shaker at room temperature and 150 rpm. The dye solution pH was 6.5. The absorbance value at 554 nm (λmax) was determined by taking certain volumes from the solution at specific time intervals and measuring the absorbance with the UV-Vis spectrophotometer. The amount of dye adsorbed on the hydrogels (q_t_, mg/g) and the dye removal efficiency (Rt, %) were calculated based on Eqs. 3 and 4, respectively. Pseudo first (Eq. 5) and second-order (Eq. 6) models were fitted to the experimental data obtained via the batch adsorption process.

(3)qt=(C0-Ct)Vm

(4)Rt(%)=C0-CtC0x100

where C_0_ is the initial concentration of RhB (mg/L), C_t_ is the concentration of RhB at the indicated time (mg/L), V is the solution volume (L) and m is the dry hydrogel mass (g).

(5)ln(qe-qt)=lnqe-k1t

(6)tqt=tqe1k2qe2

where q_e_ and q_t_ (mg/g) are the amounts of adsorbed RhB at equilibrium and specific times (t, min) and k_1_ (min^–1^) and k_2_ (g mg^–1^ min^–1^) are the rate constants of pseudo first-order and pseudo second-order kinetic models, respectively.

## 3. Results and discussion

### 3.1. Characterization of PSS based bentonite and clinoptilolite

#### 3.1.1. Natural sedimentation and zeta potential

**Figure 2:**
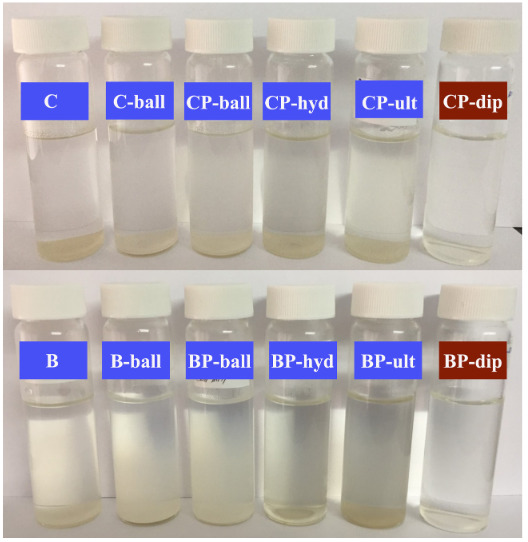
Natural sedimentation of untreated and PSS treated bentonite and clinoptilolite in ultrapure water after 3 days.

**Figure 3:**
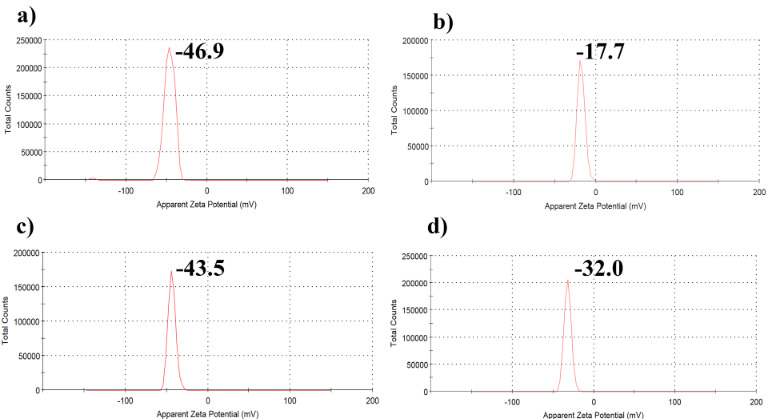
Zeta potential graphs of the inorganic fillers (a) CP-dip, (b) C, (c) BP-dip, and (d) B.

To investigate the stability and dispersibility of the PSS treated bentonite and clinoptilolite in the aqueous medium, natural sedimentation experiments were performed. The modified inorganic fillers, which were prepared by four different technique mentioned above (Table 1), and the untreated inorganic fillers were placed into glass bottles under the same conditions. The change of the stability and dispersibility within 3 days was observed by taking into account the hydrogel preparation time. It can be seen that the PSS treated bentonite and clinoptilolite prepared by the dip and dry method showed better stability and dispersibility compared to the untreated samples (Figure 2). Also, the zeta potential changes of the untreated and PSS treated fillers prepared by the dip and dry method are shown in Figure 3. When the particles have a high zeta potential (negative or positive) it means that there is a stabile dispersion medium. Thus the hydrophilic PSS added to the inorganic filler structure increased the repulsion between particles and the zeta potential. This analysis showed that the change in zeta potential value was in an affirmative manner for the PSS treated inorganic filler vis-à-vis the untreated inorganic filler. So, in the later parts of our study, the fillers which had been modified with the dip and dry method were used.

#### 3.1.2. SEM

**Figure 4:**
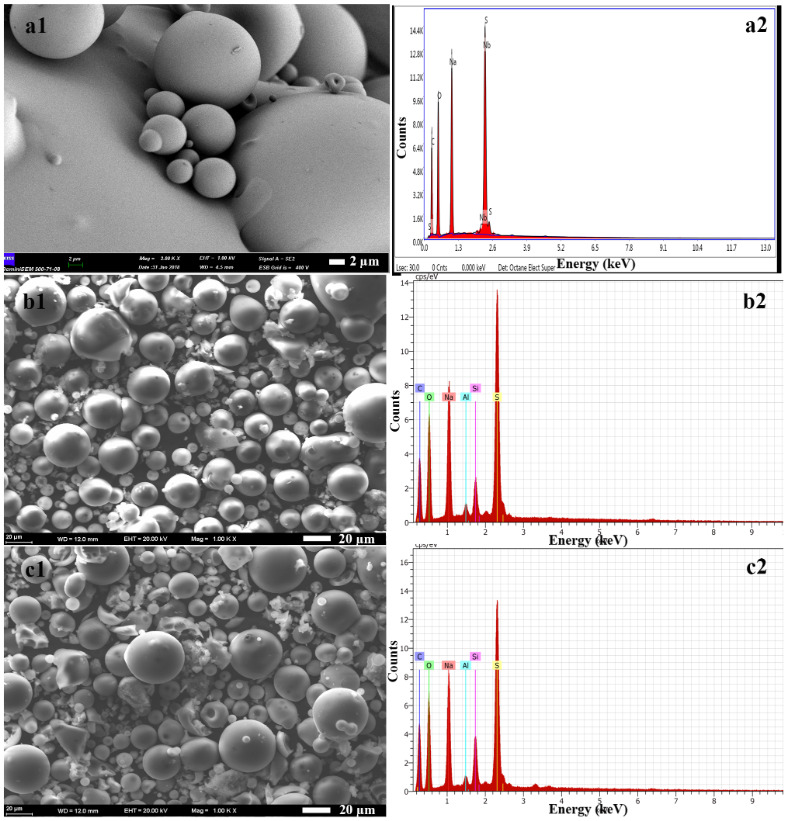
SEM photographs (1) and EDX graphs (2) of (a) PSS, (b) BP-dip, and (c) CP-dip.

As SEM photographs of the morphologies of PSS, BP-dip and CP-dip (Figures 4a1–4c1) revealed that the PSS consisted of spherical microparticles and the PSS morphology was dominant in the composite structure. The chemical compositions of the PSS, PSS modified bentonite and PSS modified clinoptilolite were analyzed by the SEM-EDX technique and the results are shown in Figures 4a2–4c2. According to the literature bentonite has two different chemical structures, Na-bentonite and Ca-bentonite. If the sodium content is predominant, the bentonite is classified as Na-bentonite, as in this study [31]. The Na, Al and Si peaks from the structure of bentonite and clinoptilolite and Na, C and S peaks from the PSS structure [25] were clearly seen in the structure of the composite fillers in Figures 4a2–4c2. The SEM and EDX results showed that bentonite and clinoptilolite were successfully modified with PSS.

#### 3.1.3. FTIR

**Figure 5:**
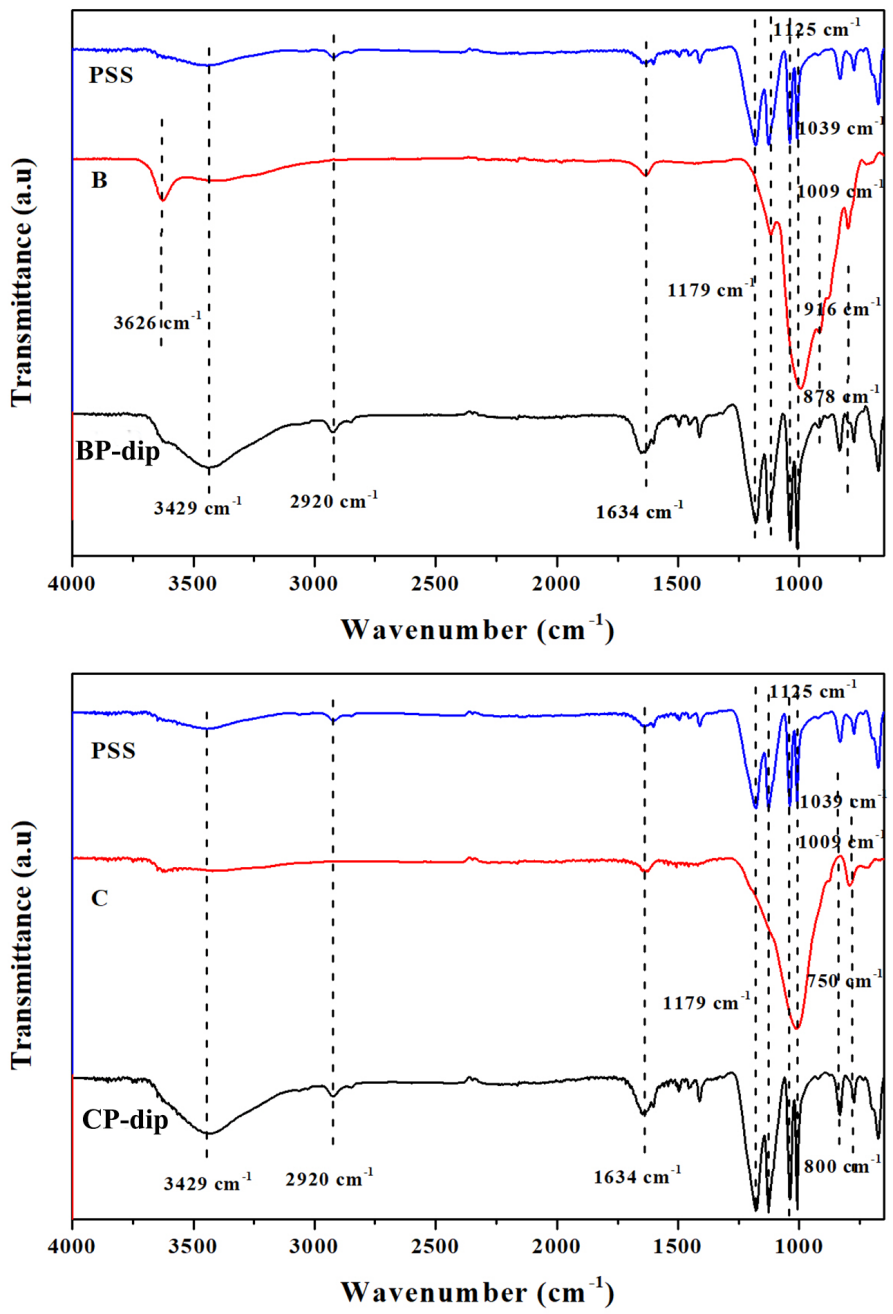
FTIR spectra of untreated and PSS treated filler prepared with the dip and dry method ((a) bentonite and (b) clinoptilolite).

Figure 5 presents the FTIR-ATR spectra of PSS, the untreated bentonite and clinoptilolite and the PSS treated ones. The absorption bands at about 1179 and 1125 cm^–1^ were attributed to the sulfonate group stretching vibrations in PSS. The peak at about 2920 cm^–1 ^was assigned to the stretching vibrations of C-H of PSS. The characteristic peaks at about 1039 and 1009 cm^–1^ are indicative of the presence of benzene ring vibrations. The bands observed between 916 cm^–1^ and 878 cm^–1^ were ascribed to the stretching vibrations of the Al-O groups from bentonite. Also, the peaks at 1634 cm^–1^ and 3626 cm^–1^ corresponded to the H-O-H and -OH stretching vibrations for bentonite, respectively [32,33]. Beside the defined peaks of PSS in CP-dip, two peaks at 750 and 800 cm^–1^ were attributed to the Si-O-Si stretching vibrations in clinoptilolite. A sharp peak in B and C, observed near 1036 cm^–1^, can be attributed to TO_4_ (T= Si or Al) stretching vibrations and this peak disappeared in the spectrum of BP-dip and CP-dip due to the much broader PSS peak at 1039 cm^–1^ [34,35]. When the characteristic bands of the inorganic fillers were compared with the peaks of PSS treated inorganic fillers, it was seen that the peaks of the treated ones had specific PSS peaks. Especially the observation of the new band at 2920 cm^–1^ in the spectra of CP-dip and BP-dip indicated that the PSS molecules were adsorbed on the filler particles [33].

### 3.2. Swelling studies and characterization of the PSS based bentonite and clinoptilolite filled composite hydrogels

#### 3.2.1. Swelling studies of composite hydrogels

To investigate the effect of the PSS treated bentonite and clinoptilolite amount on the swelling behavior of the hydrogels, the swelling studies were performed at room temperature and the results are listed in Table 3. The fillers affected the swelling degree of the hydrogels positively. This effect was due to the hydrophilic character of the PSS treated bentonite and clinoptilolite [36]. The results indicated that the addition of the PSS treated bentonite and clinoptilolite into the PVA/PVP hydrogels resulted, in general, in their bigger swelling degrees with increasing amounts of filler compared to the untreated fillers. It can be attributed to the incorporation of the PSS treated fillers into the polymer matrix, which led to higher surface area with the formation of a more porous structure [36,37]. However, due to the nonuniform dispersion of the fillers throughout the hydrogel matrix, the water intake of samples with higher filler amount was lower than that of the PVA/PVP-BP-dip25, PVA/PVP-B25 and PVA/PVP-CP-dip25/PVA/PVP-C25 hydrogels, as also shown in the studies using clay and different fillers in the literature [37,38]. Therefore, it was decided to use the composite hydrogels containing 25 mg of PSS treated fillers in the next part of the study.

**Table 3:** The swelling degree and mass loss of the bare and composite hydrogels with PSS treated filler.

Bentonite based hydrogels			Clinoptilolite based hydrogels		
Sample name	SD (%)	Mass loss (%)	Sample name	SD (%)	Mass loss (%)
PVA/PVP	265	26.14	PVA/PVP	265	26.14
PVA/PVP-B25	332	26.50	PVA/PVP-C25	353	26.20
PVA/PVP-B50	310	24.47	PVA/PVP-C50	324	25.17
PVA/PVP-B100	287	20.51	PVA/PVP-C100	292	22.51
PVA/PVP-BP-dip25	447	28.24	PVA/PVP-CP-dip25	425	28.72
PVA/PVP-BP-dip50	435	28.14	PVA/PVP-CP-dip50	418	29.74
PVA/PVP-BP-dip100	369	31.00	PVA/PVP-CP-dip100	371	29.87

Table 3 also shows the results of the polymer mass loss experiments for the bare and composite hydrogels with PSS treated filler. During and after reaching the equilibrium swelling, the mass losses occur due to the leaching of the polymer chains and fillers from the network structure [39]. By comparing the initial dry hydrogel mass with the final dried mass at the end of immersion for 15 days, the hydrogel mass loss was determined. As shown in Table 3, the PVA/PVP-CP-dip100 and PVA/PVP-BP-dip100 was the least stable hydrogel, with the filled composite hydrogels having polymer mass losses between 20.51% and 31%. Increasing the amount of PSS treated filler slightly raised the polymer mass losses of the PVA/PVP-BP-dip and PVA/PVP-CP-dip hydrogels but the polymer mass losses of the composite hydrogels were slightly decreased by using untreated fillers. This can be explained as follows: we suggest that with the inclusion of PSS the interaction strength between PVA and PVP polymers was decreased; thus a less stable network was formed with a more swollen structure. This study suggested that the PVA/PVP hydrogels were slightly more stable when filled with untreated filler compared to bare PVA/PVP hydrogel [6].

#### 3.2.2. FTIR

**Figure 6:**
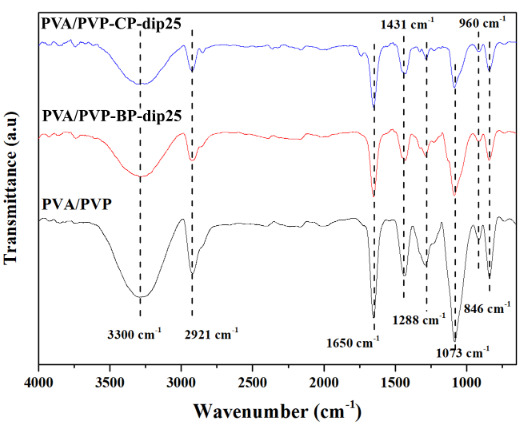
FTIR spectra of the bare and composite hydrogels with PSS treated filler.

FTIR spectra of the bare and filled composite hydrogels are presented in Figure 6. The wide and intense peaks at 3300 cm^–1^ corresponded to the -OH stretching vibrations for all of the hydrogels. The bands observed at 2921, 1431 and 1073 cm^–1^ were ascribed to the vibrations of C-H and C-O-C stretching and C-H bending for PVA, respectively [36]. The absorption bands at about 1650,1288, 960 and 846 cm^–1^ were attributed to the carbonyl stretching, C-N stretching, C-H bending of out-of-plane rings and CH_2_ bending vibrations for PVP, respectively [8,10]. No new peaks were observed in the spectrum of the composite hydrogels with PSS treated filler, since the peaks of CP-dip and BP-dip in Figure 5 are located close to the peaks of the PVA and PVP polymers and overlap with them. However, the intensity of all of the bands decreased with the incorporation of BP-dip and CP-dip in the hydrogel matrix. This change is attributed to the interaction between the polymer and the fillers [10].

#### 3.2.3. SEM

**Figure 7:**
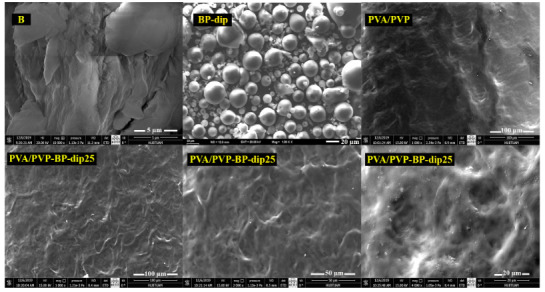
SEM photos of B, BP-dip, PVA/PVP hydrogel and the PVA/PVP-BP-dip25 hydrogel at various magnifications.

**Figure 8:**
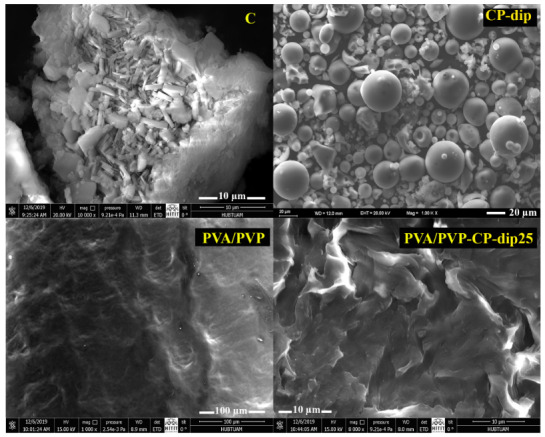
SEM photos of C, CP-dip, PVA/PVP hydrogel and the PVA/PVP-CP-dip25 hydrogel at various magnifications.

The SEM photographs of the bentonite particles/hydrogels and clinoptilolite particles/hydrogels are shown in Figures 7 and 8, respectively. The PVA/PVP image in Figures 7 and 8 showed that the hydrogel had a relatively plainer and a slightly rough solid surface with the distribution of some knolls and the B and C samples contained aggregates of their crystalline particles. The SEM images of CP-dip and BP-dip indicated that the PSS morphology was dominant in the modified filler structure. When the BP-dip and CP-dip were imbedded in the hydrogel structure, a more porous morphological structure was observed because of their homogeneous dispersion and strong interaction with the polymers. Thus, the PVA/PVP-BP-dip25 and PVA/PVP-CP-dip25 hydrogels had a rougher surface with a porous structure but absent any distinct particle dispersion.

### 3.3. Adsorption kinetics of RhB

**Figure 9:**
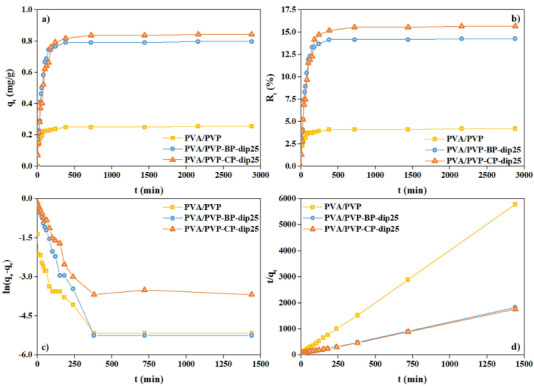
Time dependent adsorption capacity (a), dye removal efficiency (b), pseudo first-order kinetic model (c), and pseudo second-order kinetic model (d) for the hydrogels (model dye: RhB).

Adsorptions of RhB onto bare, PSS based bentonite and PSS based clinoptilolite filled composite hydrogels were investigated as a function of time by a batch adsorption method. The adsorption behavior of the bare and PSS modified bentonite and clinoptilolite based PVA/PVP hydrogels for RhB are shown in Figure 9. As can be seen in Figure 9a the time dependence for the adsorption capacity of RhB using hydrogels based on PSS modified clinoptilolite and bentonite quickly increased between 0 and 200 min due to the easy occupation by RhB molecules of the attainable sites of the composite hydrogels. The adsorption capacity slowed down as the saturation of RhB on the adsorbent occurred finally reaching an equilibrium. It is clearly seen in Figures 9a, and 9b that the filled composite hydrogels had higher adsorption capacity and dye removal efficiency than that of the bare hydrogel. The adsorption capacities were determined as 0.249, 0.791 and 0.817 mg/g for the PVA/PVP, PVA/PVP-BP-dip25 and PVA/PVP-CP-dip25, respectively. The maximum RhB adsorption capacity was 0.817 mg/g PVA/PVP-CP-dip25, which was 3.3 times higher than that of the bare PVA/PVP hydrogel. The dye removal efficiency was determined as 4.2, 14.3 and 15.7% for the PVA/PVP, PVA/PVP-BP-dip25 and PVA/PVP-CP-dip25, respectively. As can be seen, the filled composite hydrogels did not significantly affect the equilibrium time, while they increased the adsorption capacity compared to the bare PVA/PVP hydrogel. The low swelling capacity, nearly 1.6 times lower than that of the PVA/PVP-BP-dip25 and PVA/PVP-CP-dip25, of the PVA/PVP hydrogel resulted in lower RhB adsorption capacity and lower dye removal efficiency than that of the filled composite hydrogels. Bentonite and clinoptilolite increased the adsorption of the cationic RhB via electrostatic interactions because of their negative surface charge [35].

The kinetic mechanism of the RhB adsorption process was investigated at room temperature by the use of pseudo-first-order and pseudo-second-order kinetic models. The kinetic parameters of these kinetic models were obtained by using Eqs. 4 and 5, respectively. q_e_, k_1_ and k_2_ were determined from the slope and intercept of the pseudo first-order (ln(q_e_-q_t_)-t) and pseudo second-order ((t/q_t_)-t) plots, which are given in Figures 9c and 9d, respectively. The q_e_, k_1_, k_2_ and the correlation coefficients (R^2^) are listed in Table 4 for both kinetic models. As shown by the R^2^ values, the pseudo second-order kinetic model was a better fit than the pseudo first-order kinetic model for all samples. Furthermore, the calculated adsorption capacity from the pseudo second-order kinetic model was nearly the same as the experimental data for the bare and filled composite hydrogels.

**Table 4:** Kinetic parameters of the pseudo first-order and pseudo second-order kinetic models for RhB by the prepared hydrogels.

Adsorbent	Pseudo first-order kinetic			Pseudo second-order kinetic		
	qe(mg/g)	k1(min–1)	R2	qe(mg/g)	k2(g mg–1 min–1)	R2
PVA/PVP	0.085	0.0051	0.749	0.252	0.342	0.999
PVA/PVP-BP-dip25	0.435	0.0081	0.827	0.814	0.042	0.998
PVA/PVP-CP-dip25	0.520	0.0055	0.763	0.852	0.027	0.997

Considering the place of the obtained results in the literature, it was seen that comparable results were achieved. Deng et al. reported that the adsorption capacity of the PVA and SiO based hybrid hydrogel for several dyes including fluorescein, azo and other dyes from 3.49 × 10^−5^ to 1.01 × 10^−2^ and pseudo-second-order adsorption rate constant varied between 4.68 × 10^−1^ and 3.45 × 10^0^ g/mmol min [40]. Sanchez et al. studied methyl orange, methyl red and methylene blue removal with PVA-Bentonite hydrogel and stated that approximately 9%, 7% and 80% dye removal was achieved in the first 270 min by using 1% bentonite [18]. Mandal et al. studied RB adsorption of IPN hydrogel obtained by copolymerization of acrylic acid and hydroxyethylmethacrylate in PVA matrix. They were found the amount of adsorbed dye by hydrogel at about 0.08–0.12 mg/g at a maximum PVA ratio (0.5 wt %) it was reported that this value was increased up to about 0.12 mg/g after 250 min [41]. As far as we know, although no such modification has been made in the literature, considering similar studies mentioned above, it is thought that the proposed method for preparing hydrogel with inorganic fillers modified with the dip-dry technique could be contributed to the dye removal.

## 4. Conclusion

In this study a new approach was used for preparing inorganic filler to prevent their agglomeration and sedimentation during the fabrication of PVA/PVP composite hydrogels with a freeze/thawing method. For this purpose, PSS treated B and C were prepared with four methods; i.e. a hydrothermal method in an autoclave, a one-step ball milling, a dip and dry method and an ultrasonication method. For comparison, the inorganic filler and the PSS modified inorganic fillers were placed in an aqueous medium at the same time and temperature for the determination of their natural sedimentation behavior. As a result, it was shown that the BP-dip and CP-dip were successfully prepared with the use of the dip and dry method. As it is known, the dip and dry method is and simple and effective method which was used for polymer coating to nanoparticles. Based on the knowledge that hydrophilic surface modification of various materials can be made by incorporating PSS polymer to their structure, it was thought that clay derivatives could prepared as PSS modified by using this method. Eventually, the PSS treated B and C were successfully prepared according to dip and dry method. Moreover, it was found that the fillers obtained by the other methods did not show good stability and dispersibility. Also, the BP-dip and CP-dip particles had higher zeta potentials (–46.9 and –43.5 mV, respectively) than that of B and C, which means there was a stable dispersion in the medium due to the hydrophilic sulfonic acid groups on PSS. These modified inorganic materials remained stable for at least 3 days in aqueous suspension and no sedimentation was observed, with this period being sufficient for the preparation of hydrogels by the freeze/thawing method. In addition, the results obtained from the different characterization studies, FTIR, zeta potential, UV-Vis, SEM and EDX analysis, indicated that the inorganic materials were modified with the PSS and they were successfully added to the hydrogels. Swelling study results of the composite hydrogels showed that the addition of the 25 mg BP-dip and CP-dip into the PVA/PVP hydrogels provided the highest degree of swelling compared to the bare, 50 and 100 mg filled ones. The PSS treated and untreated filler content only slightly affected the solution stability of PVA/PVP hydrogels. The adsorption capacity of RhB by the PVA/PVP, PVA/PVP-BP-dip25 and PVA/PVP-CP-dip25 were evaluated. The adsorption kinetics best fitted a pseudo-second-order kinetic model. The maximum RhB adsorption capacity was determined as being nearly 2.9 and 3.3 times higher than that of PVA/PVP for the PVA/PVP-BP-dip25 and PVA/PVP-CP-dip25, respectively. As a result, it is believed that this study will contribute to the preparation of stable inorganic particles which could then be used as additives in various applications where hydrogels are used.
